# Beyond Ion Channels: Emerging Roles of FGF12 in Cellular Regulation and Cancer Progression

**DOI:** 10.3390/cells15040370

**Published:** 2026-02-19

**Authors:** Zechao Huang, Xuesen Dong

**Affiliations:** 1 The Vancouver Prostate Centre, Vancouver General Hospital, 2660 Oak Street, Vancouver, BC V6H 3Z6, Canada; 2 Department of Urologic Sciences, Faculty of Medicine, University of British Columbia, 2775 Laurel Street, Vancouver, BC V5Z 1M9, Canada

**Keywords:** fibroblast growth factor 12 (FGF12), cellular regulation, cancer progression

## Abstract

Fibroblast growth factor 12 (FGF12), a member of the intracellular fibroblast growth factor homologous factor (iFGF) subfamily, has been widely studied for its role in the modulation of voltage-gated ion channels. However, recent studies suggest that FGF12 possesses various cellular functions beyond ion channel regulation, particularly in cancer progression. Accumulating evidence indicates that the upregulation of FGF12 is associated with tumor survival, therapeutic resistance, and poor prognosis through signaling pathways independent of its canonical ion channel interactions. This review summarizes the current understanding of FGF12’s non-canonical functions, highlights its emerging roles in cellular regulation, and discusses its potential mechanism in oncogenic progression. Understanding these novel functions may provide a new aspect for therapeutic targeting of FGF12 in malignancies.

## 1. Introduction

The fibroblast growth factor (FGF) family is a large group of cell-signaling proteins that regulate a vast array of biological processes, including embryonic development, brain patterning, limb development, and tissue repair [1]. Within this family, a non-canonical subgroup known as the intracellular fibroblast growth factors (iFGFs), also referred to as fibroblast growth factor homologous factors (FHFs or the FGF11 subfamily), has attracted increasing interest [2]. Unlike canonical FGFs, which typically act in an autocrine or paracrine manner to interact with FGF receptors (FGFRs), iFGFs mainly act in an intracellular manner [3]. Fibroblast growth factor 12 (FGF12) is one of the four members in the iFGF subfamily [2]. The best-characterized function of FGF12 is its modulation of voltage-gated sodium channels [4]. Early studies demonstrated that FGF12 associates with tetrodotoxin-resistant sodium channels, thereby influencing their gating and kinetics [5]. Subsequent work has firmly established iFGFs as key regulators of cellular excitability in neurons and cardiomyocytes through direct binding to ion channels [6,7]. These ion channel-related functions are well supported by extensive biochemical, electrophysiological, and structural studies and represent the canonical biological role of FGF12.

Beyond its ion channel-related functions, emerging evidence suggests that FGF12 might participate in various cellular processes, including intracellular signaling pathways, nucleolar function, and cancer-associated regulatory networks [4]. For instance, FGF12 has been reported to interact with components of the nuclear factor-κB (NF-κB) signaling pathway [8], and multiple studies have linked FGF12 to ribosomal biogenesis [9,10]. Notably, dysregulation of FGF12 expression has been observed in several types of cancer, including esophageal squamous cell carcinoma (ESCC), gastric cancer, and others, implying a potential association with tumor progression [4]. More recently, FGF12 has been shown to be markedly upregulated in treatment-induced neuroendocrine prostate cancer (t-NEPC), a highly aggressive and therapy-resistant prostate cancer subtype, pointing toward previously unrecognized regulatory axes [11]. These findings highlight the importance of examining FGF12 from a broader biological perspective, especially in cancer studies.

Despite these observations, the mechanisms by which FGF12 contributes to cancer remain far from fully understood. Although FGF12 was long considered an exclusively intracellular protein lacking secretion signals, recent studies have challenged this view by demonstrating that FGF12 can, under certain cellular contexts, be released and interact with FGFRs to promote anti-apoptotic MEK/ERK signaling [12,13]. Nevertheless, these observations appear to be context-dependent and have been demonstrated primarily in selected experimental systems, leaving their generalizability and relevance across tumors unresolved. In addition, FGF12 exhibits a diverse subcellular distribution, including cytoplasmic, perinuclear, and nucleolar localization, and interacts with multiple protein partners ranging from ion channels to ribosome biogenesis factors [4]. How these molecular interactions are coordinated to influence oncogenic processes, such as proliferation, survival, lineage plasticity, or therapy resistance, remains an open question.

Moreover, from a translational perspective, therapeutic targeting of FGF12 also remains an unmet challenge. Current FGF-directed therapies are designed to inhibit extracellular FGF-FGFR signaling, yet such strategies are unlikely to affect intracellular, receptor-independent functions of FGF12 [14]. Emerging approaches, including small molecules, peptides, or RNA-based modalities that disrupt key FGF12 protein-protein interactions, suggest that FGF12 may be tractable through non-canonical strategies. Notably, recent studies in other iFGFs, FGF13 and FGF14, have demonstrated the feasibility of small molecules in blocking the interaction of iFGFs and sodium channels [15,16]. As a result, defining the molecular mechanisms of FGF12 in cancer is both biologically important and essential for identifying actionable targets and guiding the development of novel therapeutic strategies.

To date, no dedicated review has systematically integrated the emerging non-canonical functions of FGF12 with its potential roles in cancer biology and therapeutic targeting. In this review, we summarize current knowledge of FGF12 beyond its established role as an ion channel modulator, with particular emphasis on its functions in cancer biology. By integrating molecular, cellular, and translational evidence, we aim to provide a comprehensive framework for understanding FGF12 as a multifunctional protein with potential implications for oncogenesis, tumor progression, and therapeutic targeting.

## 2. Molecular Characteristics and Functions of FGF12

The human FGF12 gene (NCBI GenBank: NT_011651.17) is located on chromosome 3q29, as initially determined by Southern blot hybridization using rodent–human hybrid cell lines [2] and later refined by fluorescence in situ hybridization (FISH) [17]. Genomic sequencing established that FGF12 consists of five coding exons and four introns [18]. Two independent transcription start sites located upstream of exon 1 give rise to alternative first exons (1a and 1b), resulting in two major isoforms, FGF12a and FGF12b [18]. At the protein level, both isoforms adopt the conserved β-trefoil fold characteristic of the FGF family [19]. Structurally, this fold is highly similar to that of canonical secreted FGFs; however, iFGFs still functionally differ from other secreted FGFs due to the lack of the classical N-terminal nuclear localization sequence (NLS) [19]. The longer FGF12a isoform contains clusters of basic residues within exon 1a, which may function as NLS [20], while FGF12b lacks this N-terminal region and shows a more cytosolic, membrane-proximal distribution [19]. These structural differences contribute to the diverse subcellular localization patterns reported for FGF12. Functionally, FGF12b preferentially localizes to the cytoplasm, where it binds and modulates voltage-gated sodium channels. In contrast, FGF12a can be secreted out from the cells and interacts with FGFR through an unconventional manner, which involves the A1 subunit of Na+/K+ ATPase (ATP1A1) [21]. This dual localization suggests that FGF12 serves as a context-dependent signaling adaptor, integrating distinct molecular networks depending on its isoform expression and spatial distribution.

**Figure 1:**
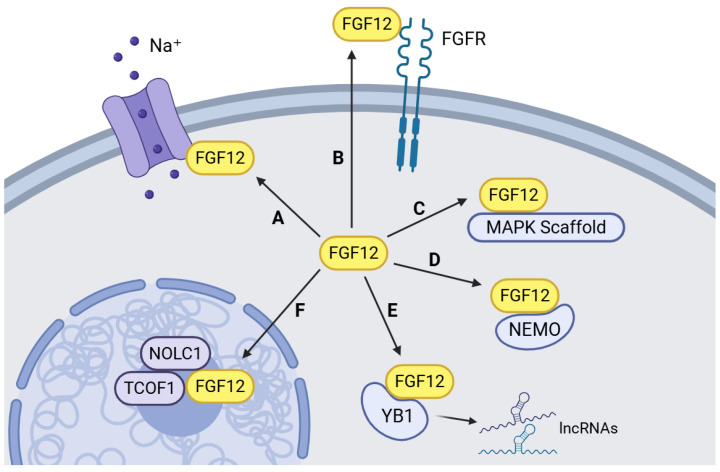
Multifunctional Roles of FGF12 in Cellular Physiology. (A) FGF12 binds directly to voltage-gated sodium channels and modulates channel gating and cellular excitability. (B) FGF12 can be released from cells and engage FGFRs to activate downstream pro-survival signaling pathways. (C) FGF12 interacts with MAPK scaffold proteins, shaping MAPK network architecture relevant to cell proliferation. (D) FGF12 associates with NEMO, altering NF-κB activation in response to cellular stress. (E) FGF12 interacts with YB1 and promotes YB1-dependent stabilization of oncogenic lncRNAs. (F) FGF12 interacts with NOLC1/TCOF1 in nucleus and nucleolus, forming complexes necessary for ribosomal biogenesis and global translational capacity. Created with BioRender.com.

The most well-studied function of FGF12 is its interactions with voltage-gated ion channels, particularly tetrodotoxin-resistant sodium channels (Figure 1 (A)) [5,6,7,22,23]. Recent work has refined its role within specific sodium channel macromolecular complexes, Nav1.2 and Nav1.6, demonstrating that FGF12 mediates sodium currents [7]. This reveals its integration into highly organized signaling assemblies that regulate neuronal excitability. In addition to electrophysiological modulation, emerging evidence also implicates FGF12 in higher-order neurobiological functions, including behavioral states associated with reward circuitry [24]. These findings expand the functional scope of FGF12 from canonical ion channel gating to broader neural network regulation.

Beyond neural network regulation, an increasing number of studies have uncovered a broader set of protein partners of FGF12, supporting its multifunctional roles in cellular regulation and cancer-relevant pathways. Early studies had demonstrated the association of FGF12 with MAPK scaffold protein, islet-brain 2 (IB2), in the adult brain, facilitating recruitment of p38 in MAPK pathway (Figure 1 (C)) [3,25]. This positions its role in stress-response and proliferation-associated kinase networks. A study later demonstrated the binding of FGF12 and NF-κB essential modulator (NEMO), a central regulator of the NF-κB signaling, enabling FGF12 to attenuate NF-κB activation and influence inflammatory or survival-associated transcriptional programs (Figure 1 (D)) [8]. More recently, proteomic analyses have expanded the functional role of FGF12 even further to its secretion and interaction with FGFR (Figure 1 (B)) [12,13,21], which was once thought to be unable before [19,26]. FGF12 is found to be secreted from cells in a context-dependent manner, which is sufficient to activate FGFR activation, initiate the downstream signaling cascade, and protect cells from apoptosis [12,13,21]. However, current extracellular FGF12 signaling was reported mainly in non-cancer or engineered experimental systems, and direct demonstration in tumor models still requires further validation. Moreover, several studies demonstrated the involvement of FGF12 in the ribosomal biogenesis process (Figure 1 (F)). FGF12 was found within nucleolar protein complexes, interacting with NOLC1 and TCOF1, two essential regulators of ribosomal biogenesis [9]. This process strictly depends on NOLC1/TCOF1 phosphorylation and requires the C-terminal region of FGF12 for their interaction and nucleolar localization [9]. In addition, galectin-1 has been shown to interact with FGF12 in the cytosol and nucleus, inhibiting its unconventional secretion and consequently affecting the assembly of FGF12-containing ribosomal biogenesis complexes [10]. This molecular interaction further highlights its multi-layered regulation role in both intracellular and extracellular spaces.

Taken together, the molecular profile of FGF12 reveals a highly pleiotropic protein that operates across multiple cellular compartments, including the plasma membrane, cytosol, nucleus, and extracellular space. Its functions stretch across regulation of ion channel dynamics, kinase signaling, inflammatory pathways, growth factor receptor activation, and ribosomal biogenesis. Such multifunctionality provides a compelling mechanistic basis for its emerging significance in cancer progression and highlights the need for deeper investigation into its tumor-associated signaling programs.

## 3. FGF12 in Cancer Biology

### 3.1. Association Between FGF12 and Cancer

Accumulating transcriptomic, proteomic, and clinical evidence indicates that FGF12 is upregulated across various human cancers, suggesting its broad oncogenic relevance (Table 1). At the transcriptomic level, multiple large-scale sequencing studies have independently highlighted FGF12 as a gene associated with poor prognosis. In diffuse large B-cell lymphoma (DLBCL), FGF12 was included as one component of a validated 14-gene prognostic signature, in which higher expression contributed to a high-risk score associated with inferior survival outcomes [27]. Similarly, in early-stage non-small cell lung cancer, a TCGA-based genomic analysis identified copy-number alterations (CNA) of FGF12 among a small set of genetic events, which were significantly associated with reduced overall survival, suggesting the potential contribution of FGF12 in early-stage lung tumor progression or aggressiveness [28]. However, in both studies, the individual prognostic independence of FGF12 was not evaluated separately from the composite models, and further studies of its clinical significance and biologic roles are required. Single-cell RNA-sequencing of low-grade endometrial stromal sarcoma (LG-ESS) further revealed selective enrichment of FGF12 in a malignant tumor subpopulation, where higher expression correlates with poor prognosis [29]. Moreover, a functional promoter variant in the FGF12 gene (rs1464938) was shown to increase bladder cancer risk in a case-control study, indicating that inherited regulatory alterations of FGF12 may also contribute to carcinogenesis [30].

Proteomic evidence further supports the relevance of FGF12 in cancer biology. Large-scale data from the Human Protein Atlas and TCGA Pan-Cancer datasets indicate that FGF12 protein expression is elevated in gliomas and renal cancers, where higher expression is associated with worse clinical outcomes, although these associations are largely descriptive and not derived from tumor-specific multivariable models [31]. In esophageal squamous cell carcinoma (ESCC), integrative analyses of TCGA and GEO datasets demonstrated that high FGF12 expression correlates strongly with advanced disease stage and poor overall survival [32]. The study further validates the high FGF12 expression by IHC staining on tissue microarrays (TMA), providing protein-level validation and strengthening its potential utility as a prognostic biomarker in this tumor type [32]. In gastric cancer, an early gene expression profiling study identified FGF12 among the genes significantly upregulated in tumor tissues compared with normal gastric mucosa [33]. Clinically, FGF12 was also included as part of a multi-analyte serum biomarker panel capable of distinguishing gastric cancer patients from healthy individuals, highlighting its promise as a non-invasive early detection marker [34]. However, this observation was based on relatively small cohorts and requires further validation in independent populations. Notably, a recent study from our laboratory demonstrated that FGF12 expression is highly upregulated at both the transcriptomic and protein levels in advanced prostate cancer states, t-NEPC [11]. By analyses through public transcriptomic datasets across different models and IHC staining with patient cohorts, the study suggested a solid upregulation of FGF12 in t-NEPC and its role in cancer cell survival and potential drug resistance [11].

Together, these findings illustrate a consistent pattern that FGF12 upregulation is frequently associated with tumor progression, aggressive phenotypes, and worse clinical outcomes across diverse cancer types. This broad oncogenic association underscores the importance of investigating FGF12 as a potential biomarker and mechanistic contributor to cancer biology.

### 3.2. Potential Mechanisms of FGF12 Function in Cancer

Although accumulating evidence suggests a strong relationship between FGF12 upregulation and the development of different types of cancer, the mechanisms of FGF12 in cancer still remain limited. At the molecular level, the longer FGF12a isoform was demonstrated to be released from cells via an unconventional secretion pathway, by the assistance of the ATP1A1 [20]. Secreted FGF12a is capable of activating FGFR signaling and downstream MEK/ERK pathways to promote cell survival in recipient cells, providing a direct route for previously thought intracellular FGFs to engage in canonical receptor tyrosine kinase signaling in the tumor microenvironment (Figure 1 B) [20]. These findings imply that, in some tumors, if FGF12 can be secreted, it may function in an autocrine or paracrine manner to enhance pro-survival signaling. Additionally, proteomic studies placed FGF12 in proximity to MAPK scaffold proteins (Figure 1 (C)) [3,23]. By interacting with MAPK scaffolds, IB2, FGF12 could locally concentrate kinases such as p38 or ERK, modulating pathway amplitude or duration and thereby influencing proliferation, stress responses, or therapy sensitivity. However, direct evidence of FGF12 to interact with the MAPK pathway in cancer are still limited, which may require future study to demonstrate.

Some other studies of FGF12 function also showed its plausible mechanisms in cancer. FGF12 binds NEMO and negatively regulates NF-κB activation in neuronal models (Figure 1 (D)) [8]. By analogy, similar interactions for FGF12 provide a plausible mechanism by which FGF12 could reprogram inflammatory and survival transcriptional outputs in cancer cells, altering cytokine responses or apoptosis thresholds. Direct evidence for FGF12-NEMO binding in tumor cells remains limited and represents an important subject for future mechanistic work. Within the nucleus, FGF12 localizes to the nucleolus and forms complexes with nucleolar assembly factors NOLC1 and TCOF1 (Figure 1 (F)). In these complexes, FGF12 appears necessary for stable assembly, linking it functionally to ribosome biogenesis [9]. Because ribosomal production and translational capacity are tightly coupled to cell growth and oncogenesis, nucleolar FGF12 has the potential to influence global protein synthesis and thus support tumor proliferation under nutrient or stress constraints. Notably, the requirement of FGF12’s C-terminal region and phosphorylation state for complex formation suggests regulatory points that could be therapeutically targeted [9].

A recent study from our laboratory expands the role of FGF12 in cancer to its association with RNA-binding proteins and long non-coding RNAs (lncRNAs) (Figure 1 (E)) [11]. We found that FGF12 interacts with the RNA-binding protein, YB1, a well-established oncoprotein involved in mRNA stabilization, translation control, and stress response [35]. The finding suggests that FGF12 can enhance the activity of YB1 in promoting the stabilization and expression of oncogenic lncRNAs, including NEAT1 and MALAT1, both of which are known to facilitate cancer cell proliferation, metastasis, and therapy resistance [35,36,37,38]. This FGF12-YB1-lncRNAs axis suggests that FGF12 could function as a post-transcriptional regulator that promotes tumor cell survival through modulation of RNA metabolism and gene expression networks.

Another key unresolved question here is the extent to which the oncogenic functions attributed to FGF12 are shared with other iFGF family members, including FGF11, FGF13, and FGF14. These proteins share high structural homology and overlapping roles in ion channel regulation, raising the possibility of functional overlap. However, current evidence suggests that their contributions to cancer progression are likely context-dependent rather than fully interchangeable. FGF12, in particular, shows distinct expression patterns in several tumor types and engages unique interaction networks, including RNA-binding proteins and ribosomal biogenesis that have not yet been reported for other iFGFs. Future work employing genetic models, isoform-specific perturbation, and proteomic interactome mapping will be required to distinguish their cancer-specific functions.

Collectively, these findings support a model in which FGF12 acts not merely as an ion channel regulation protein, but rather as a multifunctional cellular regulator with the capacity to rewrite transcriptional and post-transcriptional programs in cancer cells. Although current studies showing its roles in cancer are still limited, future studies dissecting the precise molecular interaction of FGF12 will be crucial for establishing its role as a bona fide oncogenic driver and potential therapeutic target.

## 4. Therapeutic Implications

The available data indicate that FGF12 has potential both as a biomarker and as a therapeutic target in cancer, but the evidence is still preliminary and tumor-context dependent. With respect to biomarker utility, current data primarily support a role for FGF12 as a prognostic biomarker, rather than a predictive marker of therapeutic response. As mentioned before, multiple transcriptomic and proteomic studies have reported elevated FGF12 expression associated with worse clinical outcomes in specific cancers, including ESCC, glioma, renal cancer, and advanced prostate cancer, suggesting that FGF12 expression may reflect tumor aggressiveness and biological progression. However, there is currently insufficient evidence to support the use of FGF12 as a predictive biomarker for response to specific therapies, and no studies have yet demonstrated that FGF12 expression stratifies patients according to treatment benefit. Beyond tissue-based analyses, limited evidence suggests a potential role for FGF12 as a circulating biomarker in selected cancers. In gastric cancer, FGF12 was identified as part of a multi-analyte serum biomarker panel capable of distinguishing patients from healthy controls. However, this finding was derived from relatively small cohorts and has not yet been validated in independent, large-scale studies. Furthermore, it remains unclear whether circulating FGF12 reflects tumor burden, active secretion from cancer cells, or secondary systemic responses.

From the therapeutic targeting perspective, several therapeutic strategies could plausibly be considered to exploit FGF12 biology. Because FGF12 was long considered an intracellular protein, classical extracellular approaches were not initially pursued. However, recent evidence that FGF12 can be unconventionally secreted and activate FGFR-MEK/ERK signaling opens the possibility that extracellular blockade, including FGFR inhibitors or FGF-trapping agents, could be effective [39,40]. Nevertheless, although these clinically approved therapeutic agents can engage in targeting receptor-mediated FGF12 function, they will not affect strictly intracellular functions of FGF12. For intracellular activities of FGF12, therapeutic options remain conceptual but tractable. RNA-targeting approaches, such as siRNA, could reduce FGF12 expression and are technically feasible. In addition, small molecules or peptides that disrupt critical FGF12 protein-protein interactions, such as the FGF12-YB1 interaction, could abrogate oncogenic complexes. Evidence already showed galectin-1 can bind to FGF12 in both cytosol and nucleus to block its different functions, which offers a novel angle for intervention [10]. Recent studies targeting other iFGFs, including FGF13 and FGF14, have demonstrated that several small molecules can selectively disrupt protein-protein interactions between iFGFs and voltage-gated sodium channels, providing a precedent for exploring FGF12 inhibitors through intracellular strategies beyond FGFR inhibition [15,16]. Given that FGF12 exerts many of its oncogenic effects through interaction-dependent mechanisms, targeting FGF12 protein-protein interaction networks may represent a promising and underexplored therapeutic avenue in cancer. Furthermore, targeting downstream effectors, including MAPK, NF-κB, and YB1, may offer opportunities to blunt FGF12-driven phenotypes. A recent study has shown that in non-cancer models, FGF12 can reduce stress-induced ferroptosis by activating FGFR1/MAPK/NRF2 signaling [41]. If a similar pathway is activated in cancer, small-molecule inhibitors of AMPK or modulators of NRF2 could potentially blunt FGF12’s survival benefits.

Although these therapeutic strategies are biologically plausible, there are substantial gaps and challenges for targeting FGF12 before clinical translation. First, the expression level of FGF12 could be low at early-stage cancer development. For example, in prostate cancer, the FGF12 expression is negative in hormone naïve prostate cancer. Only after cancer developed into more aggressive and lethal types, such as CRPC and t-NEPC, did the expression of FGF12 upregulated. This fact limited the treatment utility for targeting FGF12 in cancer therapy. Second, FGF12 exists as multiple isoforms and may have context-specific interactomes and subcellular localizations, complicating the selection of a universal targeting strategy. Third, because FGF12 is involved in essential processes, such as RNA metabolism and ribosome biogenesis in cells, systemic inhibition could carry toxicity risks, particularly for tissues with high baseline levels. Finally, identifying druggable interfaces, either small-molecule binding pockets or surfaces amenable to degraders, will be technically challenging and will require detailed structural and interactome mapping.

To move forward clinical application, several steps, including (1) broadened validation of FGF12 expression and prognostic value across large, well-annotated patient cohorts and multi-omics datasets, (2) mechanistic dissection of the most therapeutically relevant FGF12 interactions, such as the known FGF12-YB1-lncRNAs axis, including the structure characterization, (3) development and testing of cell-penetrant modalities, including ASOs/siRNA, small molecules, or degraders, across different in vivo models to assess efficacy and toxicity. With these steps, FGF12 could transition from an intriguing biological player to a clinically actionable target in selected cancer settings.

## 5. Conclusions

FGF12 is emerging as a multifunctional regulator in cancers, far beyond its traditional role in interacting with ion channels. Evidence from transcriptomic, proteomic, and clinical studies shows that FGF12 is upregulated across diverse malignancies and often associates with aggressive disease features. At the molecular level, FGF12 participates in several cancer-relevant processes, including intracellular signaling, nucleolar function, and RNA regulation, and may also act extracellularly through unconventional secretion. Despite these advances, the mechanisms by which FGF12 contributes to tumor progression remain incompletely understood, which poses challenges for therapeutic targeting. Moving forward, systematic mechanistic dissection and validation across cancer models are needed to define its roles and therapeutic prospect. Such efforts will determine whether FGF12 can become a meaningful therapeutic target in oncology.

## Figures and Tables

**Table 1:** Association of FGF12 and different types of cancer.

Tumors	Sources/Samples	Results/Evidences	Ref.
Diffuse large B-cell lymphoma	TCGA/GEO analyses (14 gene prognostic signature study)	High FGF12 → high-risk score and poorer survival	[27]
Non-small cell lung cancer (NSCLC)	TCGA analyses (pan-lung cancer)	High FGF12 associate with overall survival in stage I NSCLC	[28]
Low-grade endometrial stromal sarcoma (LG-ESS)	Single-cell RNA-seq (cohort study)	High FGF12 in a tumor sub-population → poor prognosis	[29]
Bladder cancer	Case-control genetic association study	FGF12 promoter variant rs1464938 → higher bladder cancer risk	[30]
Glioma/GBM	TCGA Human Protein Atlas data	Higher FGF12 in HPA/TCGA overview associate with gliomas	[31]
Renal cancer	TCGA Human Protein Atlas	Higher FGF12 in HPA/TCGA overview associate with renal cancer	[31]
Esophageal squamous cell carcinoma (ESCC)	TCGA/GEO analyses; Tissue microarray	High FGF12 → poor survival	[32]
Gastric cancer	Gene-expression reanalysis miRNA database integration	High FGF12 associate with gastric cancer patients	[33]
	Serum biomarker panel (small clinical cohort)	High FGF12 associate with gastric cancer patients	[34]
Prostate cancer	TCGA/GEO analyses; Tissue microarray	High FGF12 → t-NEPC	[11]

→: results in.

## Data Availability

No new data were created or analyzed in this study.
